# Solid Magnetoliposomes as Multi-Stimuli-Responsive Systems for Controlled Release of Doxorubicin: Assessment of Lipid Formulations

**DOI:** 10.3390/biomedicines10051207

**Published:** 2022-05-23

**Authors:** Beatriz D. Cardoso, Vanessa F. Cardoso, Senetxu Lanceros-Méndez, Elisabete M. S. Castanheira

**Affiliations:** 1 Physics Centre of Minho and Porto Universities (CF-UM-UP), University of Minho, Campus de Gualtar, 4710-057 Braga, Portugal; beatrizdiascardoso94@gmail.com (B.D.C.); vcardoso@cmems.uminho.pt (V.F.C.); lanceros@fisica.uminho.pt (S.L.-M.); 2 LaPMET—Laboratory of Physics for Materials and Emergent Technologies, University of Minho, 4710-057 Braga, Portugal; 3 CMEMS-U Minho, University of Minho, DEI, 4800-058 Guimarães, Portugal; 4 LABBELS—Associate Laboratory, 4800-122 Braga, Portugal; 5 BCMaterials—Basque Center for Materials, Applications and Nanostructures, UPV/EHU Science Park, 48940 Leioa, Spain; 6 IKERBASQUE—Basque Foundation for Science, 48009 Bilbao, Spain

**Keywords:** magnetoliposomes, stimuli-responsive, drug delivery, doxorubicin, controlled release

## Abstract

Stimuli-responsive liposomes are a class of nanocarriers whose drug release occurs, preferentially, when exposed to a specific biological environment, to an external stimulus, or both. This work is focused on the design of solid magnetoliposomes (SMLs) as lipid-based nanosystems aiming to obtain multi-stimuli-responsive vesicles for doxorubicin (DOX) controlled release in pathological areas under the action of thermal, magnetic, and pH stimuli. The effect of lipid combinations on structural, colloidal stability, and thermodynamic parameters were evaluated. The results confirmed the reproducibility for SMLs synthesis based on nine lipid formulations (combining DPPC, DSPC, CHEMS, DOPE and/or DSPE-PEG), with structural and colloidal properties suitable for biological applications. A loss of stability and thermosensitivity was observed for formulations containing dioleoylphosphatidylethanolamine (DOPE) lipid. SMLs PEGylation is an essential step to enhance both their long-term storage stability and stealth properties. DOX encapsulation (encapsulation efficiency ranging between 87% and 96%) in the bilayers lowered its pK_a_, which favors the displacement of DOX from the acyl chains to the surface when changing from alkaline to acidic pH. The release profiles demonstrated a preferential release at acidic pH, more pronounced under mimetic mild-hyperthermia conditions (42 °C). Release kinetics varied with the lipid formulation, generally demonstrating hyperthermia temperatures and acidic pH as determining factors in DOX release; PEGylation was shown to act as a diffusion barrier on the SMLs surface. The integrated assessment and characterization of SMLs allows tuning lipid formulations that best respond to the needs for specific controlled release profiles of stimuli-responsive nanosystems as a multi-functional approach to cancer targeting and therapy.

## 1. Introduction

Liposomes are biomimetic vesicles increasingly investigated in nanomedicine and pharmacology for both diagnostic and therapeutic applications [1], particularly in cancer therapy. Despite all the research and progress in the cancer field, this disease remains a significant social burden, being the first leading cause of death in people under 70 years in 112 of 183 countries [2]. The clinical failure of chemotherapy, one of the first-line cancer treatment approaches, is mainly associated with the free chemotherapeutic drugs systemic administration and the nonselective nature of the agents, leading to a reduced bioavailability at the target site [3,4,5]. Lipid-based approaches have already been shown to play an active role in the improvement of drug delivery to the tumor site while decreasing the systemic toxicity of free drugs [6,7,8]. The selectivity of these products results from a passive targeting that allows their selective accumulation in tumors (usually over 24–28 h) via the enhanced permeability and retention effect (EPR) of the leaky vasculature and the absent lymph drainage of tumors [9]. Even though the EPR effect provides a means to increase tumor specificity by 20–30% [10], it is highly dependent not only on intrinsic tumor biology (as the degree of angiogenesis and intratumor pressure), but also on the physicochemical properties of the nanocarrier (particle size, surface charge and circulation time). Thus, it becomes essential to add further targeting methods to the nanocarriers, not only to overcome the variable effect of passive targeting, but to even improve the drug delivery to the target site and, ultimately, create a synergy between different therapies.

One of the strategies used to target tumors is using thermosensitive liposomes that allow controlled release of the payload when subjected to tolerable and clinically relevant local–regional mild-hyperthermia (39–43 °C) [11,12,13]. Hyperthermia, characterized by an increase in body temperature above mean values [14], is a technique claimed to be beneficial in cancer treatment when combined with radiotherapy and/or chemotherapy [15,16]. In addition to causing tumor cells a complex apoptotic induction [17], hyperthermia allows for a 1.5 to 5-fold improvement in the radiotherapy efficiency, due, among others, to the increase in oxygenation and perfusion of cancer cells [15]. When combined with chemotherapy, it can induce an anti-cancer drug sensitization, allowing resistant cells to respond again to those drugs [18,19]. The heat leads to greater tissue perfusion and chemical reactions acceleration, enhancing chemotherapy therapeutic effectiveness [15,20]. Clinically, hyperthermia can be applied locally, regionally, or to the entire body. Hot water blankets, thermal chambers, perfusion with heated fluids, ultrasound, and electromagnetic energy are used for that purpose [21]. However, these techniques do not allow specificity for tumor cells, so they sensitize both tumor and healthy cells to the enhanced toxic effects of radio and chemotherapy. One of the most promising alternatives to ensure preferential heating of malignant cells is to use magnetic nanoparticles as mediators of magnetic hyperthermia, producing heat under the action of an alternating magnetic field (AMF) [21,22,23,24]. When combined with, for instance, thermosensitive liposomes (forming magnetoliposomes), they offer a multifunctional approach to targeting and therapy. Another strategy concerns the synthesis of pH-sensitive liposomes, whose drug release is induced by the destabilization under acidic conditions [25,26,27]. This trigger is particularly interesting in tumor targeting, as a potentiator of cellular uptake in response to enhanced acidification (in the range of 6.5–6.8) found in the extracellular tumor microenvironment resulting from oncogenic metabolisms [28]. Furthermore, endo/lysosomal pH (in the range of 5.0–6.5) induces preferential cargo release in these target cells [25,28].

In this context, the present work is focused on the design of DOX-loaded SMLs based on different lipid compositions and to evaluate the lipid combinations that validate the physicochemical, structural, and stability features for biological application in targeted cancer therapy. The main objective of studying different lipid combinations is to obtain a multi stimuli-responsive (thermo/pH sensitivity) nanosystems to enhance DOX controlled release under tumor microenvironmental and hyperthermia conditions based on (1) thermal, (2) magnetic, and (3) pH-responsiveness.

In the present work: (1) thermal-responsiveness is achieved by combining different lipids to dipalmitoylphosphatidylcholine (DPPC), which has the reversible thermotropic gel-to-liquid crystalline phase transition (T_m_ = 41.4 °C) near the temperatures used in mild hyperthermia. This feature aims the thermal-specific drug release; (2) the magnetic-responsiveness is achieved by a superparamagnetic magnetic core composed of shape-anisotropic cubic superparamagnetic nanoparticles of calcium-substituted magnesium ferrite (Ca_0.25_Mg_0.75_Fe_2_O_4_). This feature aims to (i) accumulate the DOX-loaded SMLs in tumors via a permanent magnetic field locally applied; (ii) induce magnetic hyperthermia under an alternating magnetic field for bilayer destabilization, to increase endothelial permeability to liposomes, to cause direct damage to cells, to enhance the fusion or endocytosis effect of cancer cells and to reduce the local pH of the target site [29]; (3) the pH-responsiveness is achieved by the incorporation of titrable and/or polymorphic phase lipids within the lipid bilayer, aiming a site-specific drug release in cancer tissues which, in turn, is synergistically stimulated by the warming effect caused by hyperthermia.

## 2. Materials and Methods

Ultrapure water Milli-Q grade (MilliporeSigma, St. Louis, MO, USA) and spectroscopic grade solvents were used in all the synthesis procedures. pH buffer stock solutions in the pH range between 2 and 11 were prepared from a sodium phosphate 0.1 M solution, adjusting the pH by mixing properly a solution containing citric acid (0.05 M) and boric acid (0.2 M) in ultrapure water (all reagents from Sigma-Aldrich, St. Louis, MO, USA), following [30]. The pH values were measured with a mini-pH meter NiCd-1 (Mettler-Toledo, Greifensee, Switzerland) and the pH of each solution was properly adjusted using a solution of HCl and NaOH (1 M) (all reagents from Sigma-Aldrich, St. Louis, MO, USA). For magnetoliposomes preparation, the lipids dipalmitoylphosphatidylcholine (DPPC) (from Sigma-Aldrich, St. Louis, MO, USA), distearoylphosphatidylcholine (DSPC) (from Sigma-Aldrich, St. Louis, MO, USA), dioleoylphosphatidylethanolamine (DOPE) (from Sigma-Aldrich, St. Louis, MO, USA), cholesteryl hemisuccinate (CHEMS) (from Sigma-Aldrich, St. Louis, MO, USA) and 1,2-distearoyl-*sn*-glycero-3-phosphoethanolamine-*N*-[methoxy(polyethylene glycol)-2000] (ammonium salt) (DSPE-PEG2000, from Avanti Polar Lipids, Birmingham, AL, USA) were used.

### 2.1. Solid Magnetoliposomes Preparation

**Figure 1:**
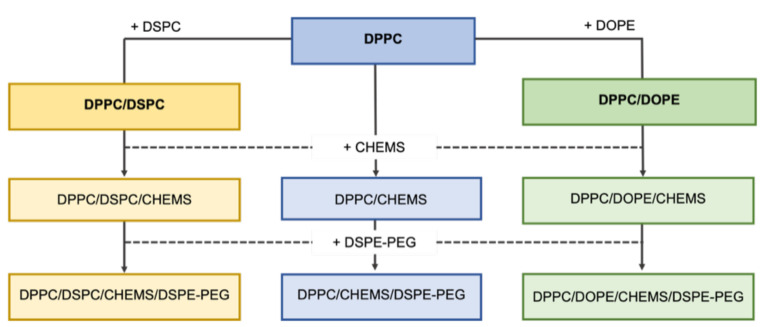
Schematic representation of the lipid combinations used to prepare DOX-loaded SMLs.

Figure 1 presents a schematic representation that summarizes the process for lipid combinations to synthesize DOX-loaded SMLs. First, the lipid DSPC and DOPE were combined to form DPPC binary formulations, branching the combinations into three groups: (A) DPPC, (B) DPPC/DSPC, and (C) DPPC/DOPE. Then, CHEMS and DSPE-PEG were sequentially added to each group, resulting in nine formulations.

The solid magnetoliposomes herein synthesized were prepared following a procedure described in [31]. All SMLs were synthesized at a total lipid concentration of 1 mM. The lipid formulations, the respective molar ratios and expected type of behavior of the SMLs are described in Table 1. First, reverse micelles were synthesized. For that, 3 mL of heptane 99% (from Sigma-Aldrich, St. Louis, MO, USA, with water content ≤ 0.1%) was added to a thin film based on the corresponding lipid formulation and ultrasonicated at a power of 190 W for a time interval for 15 min. To form a magnetic core, 1 μM of shape-anisotropic cubic superparamagnetic nanoparticles of calcium-substituted magnesium ferrite (Ca_0.25_Mg_0.75_Fe_2_O_4_, synthesized according to [31]) were added to micelles solution and subjected to 20 min of ultrasonication at the same conditions. After that, a NdFeB N48 block magnet (Eclipse Magnetics Ltd., Sheffield, UK) with nickel-plated (Ni-Cu-Ni) coating was externally applied to the sample to separate the reverse micelles with a magnetic core. The non-magnetic supernatant was discarded, and the remaining solvent was evaporated under an ultrapure nitrogen flow. The magnetic micelles were re-suspended in ultrapure water and the aqueous solution was heated at 50 °C. To form the second lipid layer, resulting in solid magnetoliposomes, an ethanolic solution containing the lipid formulation at the corresponding molar ratio (Table 1) was added, under vortexing, to the pre-heated reverse micelle solution. Additionally, a DOX ethanolic solution with a final concentration of 2 × 10^−6^ M was co-injected in this step.

### 2.2. Magnetoliposomes Characterization

#### 2.2.1. Dynamic Light Scattering Measurements

The determination of the average hydrodynamic diameter (D_H_), polydispersity index (PDI), colloidal stability and zeta-potential (ζ-potential) dependence on the pH of SMLs based on the different lipid combinations was the first approach used for the validation of nanosystems and the method to produce them for biological applications. The measurements were carried out by Dynamic Light Scattering (DLS) using a Litesizer 500 DLS equipment, possessing three detection angles (15°, 90°, 175°) from Anton Paar (Anton Paar GmbH, Graz, Austria), using a semiconductor laser diode of λ = 658 nm and 40 mW. Polystyrene cells were used to hold the samples, and a solvent refractive index of 1.33, a material refractive index of 1.4, and an absorption coefficient of 0.001 L/m were used as acquisition parameters. The polystyrene cells were washed with ethanol and then in deionized water before and after the measurements. Three independent measurements were taken for each lipid formulation, and experimental data were processed using Kalliope software (Anton Paar GmbH, Graz, Austria).

For D_H_ and PDI determination, SMLs were prepared at the corresponding molar ratio and synthesis procedure described in 2.1. Each SMLs sample was prepared with a final lipid concentration of 1 mM and filtered five times through a 0.2 μm filter before the measurements. The results are presented as mean and corresponding standard deviation from triplicate assays.

To assess the colloidal stability, SMLs were prepared at the corresponding molar ratio and synthesis procedure described in 2.1. For that, 3 mL of SMLs aqueous solutions based on the nine different lipid formulations were stored at 4 °C for 30 days at a lipid concentration of 1 mM. Changes in the D_H_ and PDI were monitored under the same acquisition and procedure conditions mentioned above.

To obtain ζ-potential dependence on the pH value and the corresponding isoelectric point, ζ-potential was measured for each lipid formulation in a pH range between 2 and 11. For that, SMLs were prepared at the corresponding molar ratio and synthesis procedure described in 2.1 and re-suspended in the buffer stock solution at the corresponding pH. The solutions were filtered five times through a 0.2 μm filter and the final pH value was measured and adjusted using a mini-pH meter NiCd-1. The results are presented as mean and corresponding standard deviation from triplicate assays.

#### 2.2.2. Fluorescence Spectroscopy Measurements

The intrinsic fluorescence of DOX [32] can be used as a facilitating tool for the characterization of the properties of DOX-loaded SMLs. Thus, the fluorescence spectroscopy technique was used to characterize the systems. Fluorescence spectra were measured in a Fluorolog 3 (HORIBA Jobin Yvon IBH Ltd., Glasgow, UK) spectrofluorimeter, equipped with Glan-Thompson polarizers and double monochromators in excitation and emission. Fluorescence spectra were corrected for the instrumental response of the system. The excitation of the DOX-loaded SMLs was set at λ_exc_ = 480 nm and the emission spectrum was collected between 490 nm and 650 nm, with a slit of 4 nm in both excitation and emission.

Fluorescence emission measurements were performed to quantify the encapsulation efficiency, EE%, of DOX in SMLs based on the different lipid formulations. After preparation, drug-loaded SMLs were placed in Amicon^®^ Ultra-0.5 mL centrifugal filters with 0.1 μm pore size and centrifuged at 3000 rpm for 10 min. The supernatant was pipetted out and its fluorescence was measured, allowing to determine the drug concentration using a calibration curve previously obtained. Three independent measurements were performed for each system and standard deviations (s.d.) were calculated. The EE% was determined using Equation (1).

(1) $$EE\%=\frac{Initial\;drug\;concentration-drug\;concentration\;in\;the\;supernatant}{Initial\;drug\;concentration}\times 100$$

Steady-state anisotropy fluorescence values, r, are dependent on the fluorophore rotational diffusion (and thus dependent on viscosity, temperature, and molecular size of the fluorophore) [33] that can be used to study drug-lipid vesicle interactions. The steady-state fluorescence anisotropy, *r*, was measured in the latter equipment, using Glan–Thompson polarizers, and an average value in an appropriate spectral range was calculated by Equation (2) [34],

(2) $$r=\frac{I_{VV}-GI_{VH}}{I_{VV}+2GI_{VH}}$$

where $I_{VV}$ and $I_{VH}$ are the intensities of the emission spectra obtained with vertical and horizontal polarization, respectively (for vertically polarized excitation light), and *G* = $\frac{I_{HV}}{I_{HH}}$ is the instrument correction factor, where $I_{HV}$ and $I_{HH}$ are the emission intensities obtained with vertical and horizontal polarization (for horizontally polarized excitation light). Several assays were performed for the different lipid formulations over a range of pH from 2 to 11, to associate DOX rotational mobility with pH variation.

Human serum albumin (HSA) is the most abundant protein in human plasma that, by interacting with drugs, influences their pharmacokinetics and pharmacodynamics [35]. The intrinsic fluorescence of HSA allows the drug/protein interaction study by fluorescence spectroscopy, through the analysis of the fluorescence quenching that results from that interaction [35,36]. The interaction of DOX-loaded liposomes with HSA was studied following the same procedure described in the previous work [31]. An aqueous solution of HSA of a fixed concentration of 0.2 mM (mimicking the HSA blood plasma concentration) was titrated with DOX-loaded liposomes. For that, 1 μL of liposomes were added between each increment, and the sample was left stabilizing at room temperature for 20 min. The excitation of tryptophan residues was set at λ_exc_ = 280 nm, and the emission spectra were recorded in the range of 290–700 nm, with an integration time of 1 s and the width of the slits set to 2 nm. The changes in the maximum fluorescence emission intensity found at 344 nm was analyzed, and the relative efficiency of HSA quenching can be described by Equation (3) [36],

(3) $$\%\;quenching=\frac{y_{max}^{n}}{1+\frac{k_{d}}{\left[ligand\right]}}$$

where $y_{max}$ is the maximum fluorescence quenching registered, n is the number of binding sites, and $k_{d}$ is the dissociation constant. The affinity between the protein and the ligand ($k_{b}$) is inversely proportional to $k_{d}$ and can be expressed by 1/$k_{d}$.

The effect of different lipid combinations on DOX release kinetics was quantified for different environments and conditions, in order to evaluate the effect of thermo/pH-sensitive lipids combination in multi-stimuli-responsive SMLs. Thus, a reusable 96-well Micro Equilibrium Dialysis Device, HTD 96b from HTDialysis, LLC (Gales Ferry, CT, USA) with regenerated cellulose dialysis membranes, was used to assess DOX release kinetics profile from the different lipid compositions. The assays were performed at pH 5.5 and 7.4 to simulate the drug release profile in the acidic tumor microenvironment and physiological fluids, respectively [27,28]. They were also carried out at 37 °C and 42 °C, representing physiological temperature and hyperthermia conditions [11,13]. The DOX release was followed for 30 h by collecting the samples from the acceptor compartments at different time points and measured by fluorescence spectroscopy (λ_exc_ = 480 nm, in the range of 550–650 nm). The experimental DOX release profiles were fitted to different kinetics models (Weibull [37], first-order [38] and Korsmeyer-Peppas [39]—see Supplementary Materials) using Prism 8 software (GraphPad Software, La Jolla, CA, USA).

#### 2.2.3. Differential Scanning Calorimetry

Differential scanning calorimetry (DSC) measurements were performed to determine the temperature transition from gel to liquid-crystalline (T_m_) of DOX-loaded SMLs and, thus, evaluate their potential as thermosensitive nanocarriers. The measurements were performed in a Mettler-Toledo DSC822e apparatus with Sample Robot TS 0801 RO (Mettler-Toledo, Columbus, OH, USA). First, 30 μL of the prepared SMLs samples (1 mM) were placed into the corresponding 40 µL aluminum pans, accurately weighted on an analytical balance, and sealed. Each sample was placed in the calorimeter and isothermally held at 25 °C for 5 min before heating to 60 °C, with a scanning rate of 2 °C/min. Three independent measurements were performed for each system, and all experiments were carried out under a nitrogen atmosphere at a temperature between 25 and 60 °C. The peak temperature (T_m_) and the width of the main peak were determined from the DSC curve.

## 3. Results and Discussion

### 3.1. Influence of Formulation on Structural, Colloidal and Thermodynamic Parameters of DOX-Loaded SMLs

In this work, DPPC was selected as the primary lipid for every lipid formulations, due to its drug encapsulation capacity, thermosensitivity, and because it was already validated as suitable for biomedical applications and controlled release [31]. However, it is reported that drug release occurs slightly before the main transition temperature peak [40], the rate and amount of drug release being relatively low. As DSPC has a higher transition temperature (T_m_ ≈ 55 °C), the binary formulation of DPPC/DSPC mixture results in more rigid vesicles, associated with a reduction in their clearance by the mononuclear phagocyte system (MPS) [41,42], while enhancing the amount of drug release [42,43]. Furthermore, it is reported that the amount of induced nanoscale gaps in liposome membranes can be adjusted by changing the ratio between DPPC and DSPC to enhance the release kinetics [44]. The neutral cone-shaped lipid dioleoylphosphatidylethanolamine (DOPE) was used to synthesize pH-sensitive SMLs. Due to its conical shape and its packing parameter higher than 1, this lipid forms non-bilayer structures at physiological pH. Cholesteryl hemisuccinate (CHEMS), a weakly acidic amphiphile, is commonly used as a complementary molecule to stabilize DOPE bilayers at physiological pH by filling the space between the head groups of DOPE. The pH-induced cargo release mechanism relies on destabilization caused by the transition from a bilayer structure (L_α_ phase) at neutral pH to the inverted hexagonal phase II (H_II_ phase) at acidic pH [45]. CHEMS can reduce acyl chain mobility of DPPC above the phase transition [46], its protonated form mimics some of the membrane properties of cholesterol [47] while acting as a DPPC membrane stabilizer [46]. Furthermore, the incorporation of CHEMS in lipid formulations based on DPPC and/or DSPC has already proven to increase the interaction of liposomes with target cells (with negative surface charge) and, in addition, make the vesicles more fusogenic when exposed to acidic pH (as in endosomes and lysosomes) [48]. Polyethylene glycol (PEG), which steric hindrance prevents their aggregation and reduces the absorption of plasma proteins, was added to the lipid formulations because of the reduced stability and half-life in the bloodstream of conventional liposomes [49].

Evaluating the average size, PDI, and stability of nanosystems is typically the first most relevant step in validating a lipid-based nanocarrier as a drug delivery system and the method to produce it. The vesicles size significantly influences, for example, pharmacokinetics, tissue diffusion, and kidney excretion, so its determination makes it possible to predict some of the system’s behavior [50]. It is generally accepted that the desirable size of drug delivery systems should be between 50 and 200 nm [51]. In addition, PDI values allow relating the degree of heterogeneity of size distributions, and it is recognized that, for this type of application, the PDI values must be equal or below 0.3, indicating a homogeneous population of the systems [50]. The combined analysis of these parameters is one of the criteria used to assess the stability of the systems, either in the short, medium, and long term. The results of DLS measurements are summarized in Table 2. All the studied lipid formulations revealed a hydrodynamic diameter below 200 nm. In every groups, it was noticed a negligible effect in size of CHEMS and a greater increase when DSPE-PEG was added. The lipid formulations revealed a size narrowly distributed, with a polydispersity index below the 0.3 limit. It is thus demonstrated that all lipid formulations resulted in DOX-loaded SMLs with adequate sizes and PDI to act as drug delivery systems.

The drug encapsulation in lipid vesicles can be associated with the synthesis method for production, the lipid composition, and the drug itself [52]. DOX encapsulation efficiencies (EE%) were determined by the percentage of incorporated drug into SMLs relative to the initial amount of drug added (Equation (1)) and the results are also summarized in Table 2. All formulations demonstrated a high drug encapsulation efficiency, ranging between 87% and 96%, which allows us to conclude that the different lipid formulations do not significantly influence encapsulation at this drug/lipid ratio. Furthermore, it proves the reproducibly of the methodology previously developed for other lipid formulations [31].

**Figure 2:**
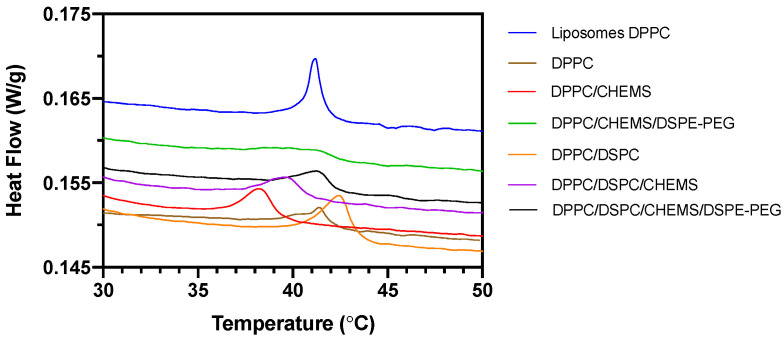
DSC heating thermograms of DOX-loaded SMLs based on different lipid formulations.

DSC measurements assessed their potential as nanocarriers for controlled drug release under hyperthermia conditions by determining the temperature transition from gel to liquid-crystalline (T_m_). Figure 2 summarizes the average DSC scans of the three independent measurements performed for each system. Initially, the DSC curve for pure DPPC-based liposomes was obtained to study the influence of magnetic nanoparticles on DSC curves. A decrease in the intensity of the transition temperature peak and the appearance of a double transition peak was noticed in SMLs compared to the liposomes counterparts. Drazenovic et al. [53] described similar results as a direct effect of the lamellarity and size. The authors found that when subjecting large unilamellar DPPC vesicles (LUVs) to the extrusion process through sequentially lower polycarbonate filters (400 nm, 200 nm, 100 nm, and 50 nm), the intensity and definition of the peak gradually decreased and a second peak appeared and broadened with decreasing vesicle sizes. Our DSC results may have influence of size and of the presence of magnetic nanoparticles. The remaining lipid combinations demonstrate a similar peak broadening pattern, which is justified not only by those effects mentioned above, but also because they are lipid mixtures rather than single-component lipid systems. Furthermore, this peak broadening is associated with an ideal mixing of lipids acyl chains [54], as well as the lowering of cooperativity of the phase transition [55].

The thermograms corresponding to Group C are not shown in Figure 2, since it was not possible to record any phase transition in these formulations. These results are due to the presence of DOPE (T_m_ ≈ −16 °C), which results in lipid combinations with a T_m_ lower than the detection limits programmed in the experiment (limits of interest in hyperthermia conditions).

The calculated T_m_ for each formulation is also shown in Table 2. DPPC-based SMLs show a similar transition temperature (T_m_ = 41.40 ± 0.02 °C), to that obtained for the liposomes (T_m_ = 41.1 ± 0.15 °C). The addition of CHEMS to those SMLs resulted in a decrease of T_m_ to 38.2 ± 0.2 °C. The subsequent addition of DSPE-PEG raised the T_m_ to 41.34 ± 0.02 °C, which is a similar value to that of the base formulation.

Concerning Group B, the addition of DSPC to the lipid base of DPPC raised the T_m_ to 42.4 ± 0.2 °C, which was expected since DSPC has a T_m_ of ~55 °C. This result indicates that the binary DPPC/DSPC mixture allows obtaining systems with a tunable T_m_ (varying the ratio of both lipids), offering the system the practical advantages of both lipids. Similar to Group A, the addition of CHEMS to the DPPC/DSPC base formulation resulted in a decrease of T_m_ to 39.7 ± 0.1 °C. This deviation was mitigated by adding DSPE-PEG to the latter, which slightly increased T_m_ to 41.4 ± 0.2 °C. This effect, resulting from the increasing inclusion mol% of DS chains of DSPE-PEG, has been previously described by Needham et al. [56].

The thermosensitivity of the formulations in Groups A and B was confirmed. Further, the most complex lipid formulations (the PEGylated ones) assume an optimal thermosensitivity for controlled drug release under hyperthermia conditions. Even though the structural characterization techniques validated the primary requirements of the nine lipid combinations for the proposed application, the thermodynamic parameters only allow the validation of the formulations of groups A and B as thermosensitive formulations.

**Figure 3:**
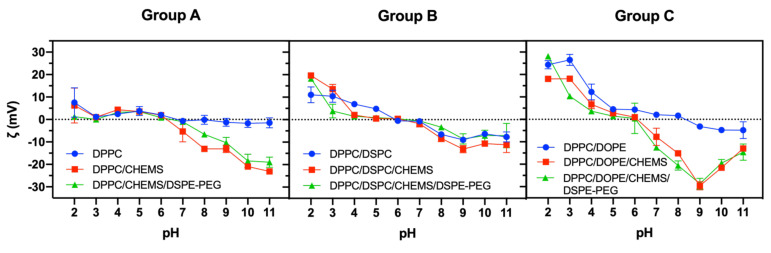
ζ-potential-pH profiles of DOX-loaded SMLs based on Group A, Group B and Group C lipid formulations.

The ζ-potential dependence on pH of aqueous DOX-loaded SMLs was measured at pH values ranging from 2 to 11 (Figure 3). In general, the ζ-potential of SMLs changed from negative to positive with decreasing pH values. This behavior is beneficial to potentiate the electrostatic interaction between magnetoliposomes and tumor tissues [57]; the latter has acid-outside plasma pH gradients, while normal tissues have an alkaline one [57]. However, large variations of ζ-potential values were not observed in any of the binary formulations of each group (DPPC, DPPC/DSPC, and DPPC/DOPE) in the pH window of therapeutic interest (± between 5 and 8), the formulations showing a near-neutral ζ-potential, as commonly observed for formulations based on zwitterionic lipids. It is noted that the addition of CHEMS to the formulations alters their ζ-potential, which is more relevant in Group A and C. In these groups, the absolute values of ζ-potential increased compared to the base formulation, being more significant for pH higher than 7. The estimated pH at the isoelectric point was calculated and is presented in Table 2. In all groups, there was a reduction of the pH of the isoelectric point with the addition of CHEMS; this behavior was maintained, although for slightly higher values, with the subsequent addition of DSPE-PEG.

Due to its protonable amino group, DOX is positively charged at pH 7. It was found that most formulations showed negative ζ-potential values at pH 7, except for DPPC/DOPE formulations whose estimated ζ-potential was slightly positive (+2.07 mV). Furthermore, by masking the positive charge of DOX, these systems are a vehicle to avoid multi-drug resistance (MDR) that limits the clinical success of chemotherapy [57]. In fact, the overexpression of some plasma proteins on the surface of tumor cells—such as membrane P-glycoproteins that can extrude positively charged compounds—prevents the intracellular accumulation of drugs and their therapeutic action [57,58].

### 3.2. Interaction with HSA

**Figure 4:**
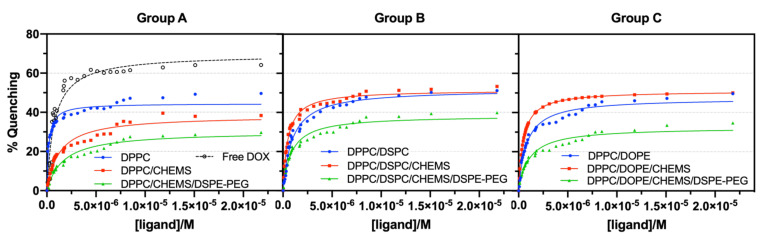
HSA fluorescence quenching (%) as a function of increasing free DOX concentration (as a reference in Group A) and loaded in Group A, Group B and Group C lipid formulations. All experiments were performed at pH 7.4 and room temperature. A non-linear fit according to Equation (3) is presented (corresponding lines).

It is also essential to insight the stability of formulations under physiological conditions, which will significantly determine the bioavailability of drugs on target site. The interaction of nanosystems with plasma proteins, such as the human serum albumin (HSA), is one of the indirect techniques studied for this purpose. These assays are based on the fluorescence quenching effect associated with the Trp214 residue (located in the hydrophobic cavity of the HSA) that results from changes in the conformation of HSA when binding to lipid vesicles [36]. In practical terms, the lower the degree of interaction between the lipid vesicles and HSA, the smaller the associated fluorescence quenching effect. Figure 4 summarizes the results obtained for each group of formulations, presented as HSA fluorescence quenching (%) as a function of DOX-loaded liposomes increasing concentration. The non-linear fit, following Equation (3), is also shown, and the calculated variables (dissociation constant, binding constant, and the number of binding locations) are presented in Table S1 (see Supplementary Materials).

The considerably higher binding constant for free DOX compared to that obtained after its encapsulation in lipid systems reveals the capacity of the latter to provide effective drug protection against interaction with HSA, preventing opsonization and increasing its bioavailability. Group A (DPPC-based) revealed a decrease in affinity for HSA when CHEMS was added to the formulation (Figure 4). Group B (DPPC/DSPC) shows, in general, a slightly higher binding affinity to HSA compared to Group A. Semple et al. [59] reported that DSPC liposomes bind more to serum proteins than DPPC liposomes, in accordance with our results. The inclusion of CHEMS in the lipid formulation induces slightly higher binding affinity to HSA than that obtained for systems without CHEMS. DPPC/DSPC/CHEMS systems revealed twice the binding constant value (k_d_ = 2.12 × 10^6^ M^−1^) compared to the DPPC/DSPC formulation (k_d_ = 1.01 × 10^6^ M^−1^), with a very similar number of binding locations (1.66 and 1.67, respectively). Since CHEMS is a cholesterol (Ch) mimicking molecule, and following the results of Semple et al. [59], a decrease in protein binding ability was expected. The authors demonstrated that an increase in Ch content (up to 30 mol% Ch) caused a significant reduction in protein binding to the vesicles. This effect come from the ability of Ch to alleviate packing defects (angular fracture planes) characteristic of DSPC-based large unilamellar vesicles (LUVs) and to which serum proteins are more easily adsorbed. However, these results may be merely size related, as these defect regions are common in LUVs, which are considerably larger than the SMLs studied here. A similar behavior was also found in Group C (DPPC/DOPE-based systems).

All the PEGylated nanosystems revealed enhanced stealth properties within the corresponding group. Following those obtained in the previous section, these results allow us to conclude that the inclusion of a small amount of DSPE-PEG provides greater long-term storage stability and cargo protection under physiological conditions. The binding of HSA results from the electrostatic interaction between the anionic residues of HSA and the cationic choline lipid head groups [60]. It is also reported that the degree of binding of HSA to PEGylated bilayers depends on PEG molecular weight and grafting density, as well as PEG conformation in the vesicles. When PEG has a mushroom conformation, HSA can penetrate the PEG layer and bind to the bilayer, which does not happen in a brush state [60]. Considering the low %mol of DSPE-PEG2000 added to the lipid formulations, and according to the simulations by Lee and Larson [60], PEG assumes a “mushroom” regime. In this regime, PEG has a conformation similar to a single chain isolated in water rather than a dense layer, which justifies the electrostatic binding of HSA to the bilayer and the overcome of the PEG steric effect.

### 3.3. Stability of Liposomal Aqueous Suspensions upon Storage at 4 °C

**Figure 5:**
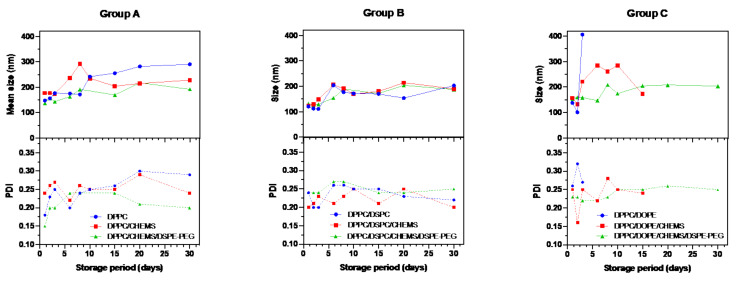
Stability data of DOX-loaded SMLs aqueous suspensions, based on Group A, Group B and Group C lipid formulations, upon storage at 4 °C. The stability of the formulations is expressed as the mean size and PDI variations compared to the original values (measured at day 1).

The stability of magnetoliposomes was monitored for 30 days by DLS measuring he variation of system size and size distribution compared to day 1 values. The storage method at 4 °C, is the most common storage method for lipid vesicles in aqueous media [61]. Groups A and B (DPPC and DPPC/DSPC as the base formulation, respectively) stand out as those that showed minor variations in size and PDI after 30 days of storage (Figure 5). It has been described that spherical particle with sizes between 50 and 200 nm can more easily avoid uptake by liver and spleen, and avoid kidney clearance exhibiting a greater tendency for long circulation [62]. Therefore, the 200 nm size is used in this analysis as the stability limit of the systems.

In Group A, it was found that the addition of CHEMS to DPPC caused the SMLs to exceed the 200 nm limit at day 6, whereas in the pure DPPC formulation, this mark was only exceeded at the 10th day. These results may be related to the decrease in the transition temperature also observed in these systems. The stability of lipid systems is dependent, for instance, on the effects of lipid chains length and the transition temperature associated to lipid vesicles [63]. Longer acyl chains result in stronger van der Waals interactions, resulting in a transition temperature increase. Prislan et al. [64] demonstrated that longer acyl chains increase the stability of lipid bilayers. Likewise, the rate of hydrolysis tends to increase with decreasing chain length of phospholipids [65]. The DPPC/CHEMS/DSPE-PEG formulation has the smallest size variation over time and, therefore, the greatest stability. The 200 nm limit was only exceeded on day 20 while the PDI values remained reduced, not exceeding the 0.3 threshold. The behavior of the systems in Group B remains, in general, very similar among all the formulations. The formulations recorded a peak size increase around day 5, less pronounced in the DPPC/DSPC/CHEMS/DSPE-PEG formulation, followed by a period of stabilization. The PDI of these formulations showed similar behavior, always maintaining adequate values for the application. Regarding Group C, only the PEGylated formulation demonstrated stability. In contrast, the DPPC/DOPE formulation duplicate the size at day 3, with the appearance of multiple-sized populations and, as such, a significant increase in PDI. The measurements in this formulation were interrupted on day 8, due to the formation of polydisperse aggregates, with size values larger than 600 nm. The addition of CHEMS to the DPPC/DSPC formulation helped to stabilize the membranes; however, from day 5 onwards, the sizes were not adequate, and from day 10 onwards, the samples presented multiple population sizes (which made it impossible to continue the measurements). The PEGylated formulation was the only one that could be measured up to day 30. In general, the effect of adding a small amount of DSPE-PEG as a long-term stabilizer of all lipid formulations when stored at 4 °C is highlighted.

### 3.4. Implications of Lipid Formulation and pH on Location of DOX

Fluorescence spectroscopy and steady-state fluorescence anisotropy measurements of DOX-loaded SMLs were performed in a pH range between 2 and 11. This assay had as main objectives: (i) to associate fluorescence intensity with the distance of DOX from the magnetic core with pH variation; (ii) to connect DOX fluorescence anisotropy to its rotational mobility; and (iii) relate the combined effect of pH and lipid composition to the location of DOX in the lipid systems and predict the ease of DOX release in the studied pH range.

**Figure 6:**
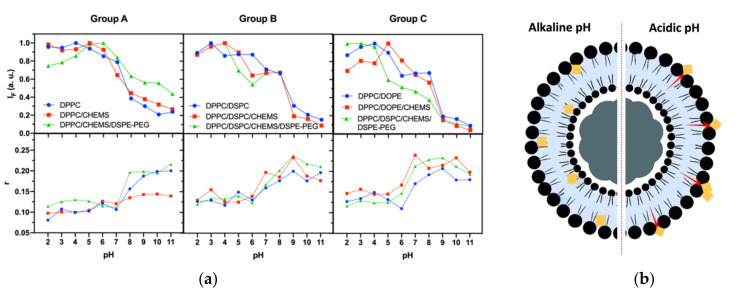
(**a**) Fluorescence intensity and steady-state fluorescence anisotropy (r) variation with pH (from 2 to 11) of DOX-loaded SMLs based on different lipid formulations. Total lipid concentration: 1 mM; DOX: 2 µM. (**b**) Schematic representation of DOX molecules localization in the lipid bilayer at different pH (predictive for formulations with DPPC as the base lipid).

The analysis of fluorescence intensity changes is based on the distance-dependent quenching effect exerted by magnetic nanoparticles in DOX fluorescence; however, it must also be considered that the fluorescence emission of DOX presents a mild pH-sensitivity (<5% per pH unit) [41]. The steady-state fluorescence anisotropy values (obtained at 25 °C using Equation (2)) reflect DOX location, considering the fluidity decrease from the bilayer surface to the interior, where the acyl chain end shows increased disorder [66]. The analysis of fluorescence intensity (I_F_) variations must also be supported by the anisotropy results at the corresponding pH. Figure 6a shows a decrease in I_F_ with increasing pH, which can be explained by DOX pH-sensitivity and its distance to the magnetic core (that acts as a quencher). The abrupt drop recorded in all formulations together with the increased anisotropy, proves the formation of magnetoliposomes and the DOX incorporation into the nanosystems.

Considering that DOX has pK_a_ ≈ 9.93, the pronounced variation of I_F_ and anisotropy for pH between 5 and 7 again proves the incorporation of DOX into the lipid bilayer, as its neutral form is favored. In the case of basic groups (like DOX), a tendency to shift pK_a_ to values below the normal values in water is reported [67,68], which is verified for DOX in a hydrophobic environment (such as lipid bilayers). The obtained anisotropy values are consistent with the typical values for fluorophores in bilayers and lower than the fundamental anisotropy reported for DOX (r_0_ = 0.33) [43].

Similar I_F_ and anisotropy variation profiles were found in all lipid formulations. Those profiles are characterized by a general decreasing trend in I_F_ with increasing pH and an inverse effect in anisotropy values. Figure 6b shows a schematic representation (predicted by the obtained results) of DOX position in the lipid bilayer when subjected to different pH conditions. At alkaline pH, DOX is near acyl chains (higher anisotropy and lower I_F_ values) and, in acidic media, it moves to the membrane surface, even being adsorbed on the surface of SMLs. These results allow predicting a higher ease of DOX release at lower pH, namely at pH 7 for Group A formulations and pH 6 for Groups B and C. Furthermore, it is also verified that the functionalization with DSPE-PEG also allows the protonation of DOX and, as such, its location on the surface of the SMLs. However, less release is expected, since PEG acts as a barrier on drug diffusion.

### 3.5. Effect of Lipid Formulations on DOX Release Kinetics and Profile

**Figure 7:**
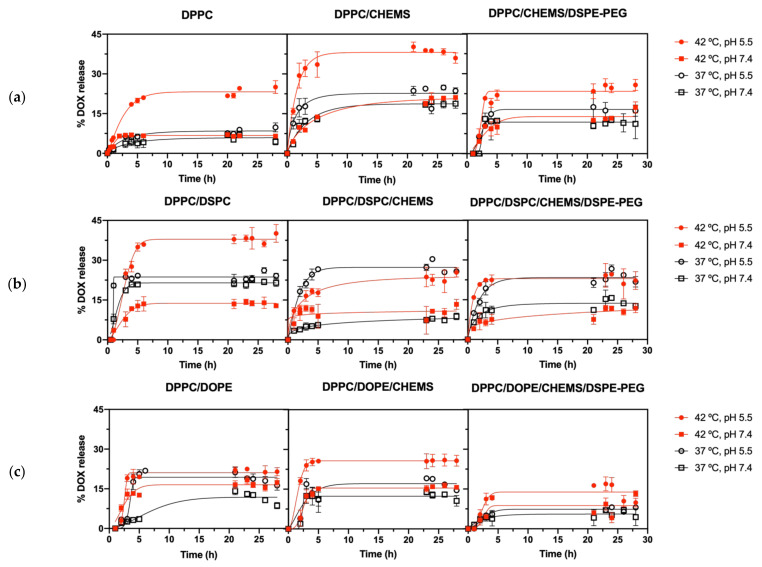
In vitro kinetic release profile of DOX encapsulated in SMLs based on (**a**) Group A, (**b**) Group B, and (**c**) Group C lipid formulations at different conditions of temperature and pH. Triplicate mean fitted to the Weibull kinetic model.

DOX release assays from the nine SMLs formulations were carried out in different environments and conditions. A lipid formulation suitable for controlled release in cancer therapy should potentiate DOX release at therapeutic conditions (42 °C and/or acidic pH), while reducing non-specific release under physiological conditions. Additionally, it should not present a very pronounced release over the first 24 h. These assays measured DOX release using as triggers the combined effect of temperature and pH (Figure 7). These screenings intend to pre-select the lipid formulations that best fit controlled release patterns to a future exploration using an alternating magnetic field as a trigger.

Table 3 shows the ratio between the maximum release value for each formulation and the one obtained under physiological conditions (37 °C, pH = 7.4). It is intended to compare the interplay of the thermosensitivity with the pH sensitivity. The slope (*m*) of the release plotted in the first 5 h was calculated for a better interpretation of the short-term response upon stimuli application. Therefore, multi-stimuli-responsive formulations for DOX controlled-release should present: (i) a release ratio > 1 at acidic pH, related to the ability to release DOX locally in tumors; and (ii) a higher release rate at 42 °C, pH 5.5 than that obtained at 37 °C, to ensure a local temperature-triggered controlled release.

Concerning Group A, there was a general increase in DOX release with the addition of CHEMS to the DPPC formulation. Besides the evident increase in maximum release at 42 °C and pH 5.5 (ideal therapeutic conditions), there is an accentuation of the release at 37 °C, pH 5.5 in the first hours. There was not a significant variation in the release profiles at pH 7.4. These results point to a synergy between pH and temperature stimuli, which are determining factors in the release of DOX, and also reflect the pH-dependent solubility of DOX due to the protonation of anthracycline moiety at acidic pH. A similar profile was found upon the addition of DSPE-PEG to the latter (DPPC/CHEMS/DSPE-PEG) formulation. However, the obtained maximum release of DOX was lower than the formulation without DSPE-PEG. The same behavior was reported previously [31] and may be associated with a diffusion barrier action of PEG on the surface of SMLs.

The addition of DSPC to the DPPC formulation (Group B) was shown to generally potentiate DOX release. The DPPC/DSPC binary formulation allowed a 1.6× increase in the total DOX released and a higher release rate in the first 5 h. Once again, an acidic pH-determining action is noted, even under physiological temperature conditions, resulting from drug protonation. At pH 7.4, the release profile is very similar. Unlike the Group A counterpart, the addition of CHEMS reduced the maximum DOX release, except at 37 °C and pH 5.5. This may result from a prevalence of pH sensitivity over thermosensitivity, as the addition of CHEMS reduced T_m_ from 42.4 °C to 39.7 °C. The addition of DSPE-PEG (DPPC/DSPC/CHEMS/DSPE-PEG formulation) led to an increase in T_m_ to 41.4 °C and resulted in a slight decrease in the maximum release at 37 °C and pH 5.5. It should be noted that the SMLs based on DPPC/DSPC/CHEMS/DSPE-PEG were the ones that showed not only a higher release rate in the first 5 h at 42 °C and pH 5.5, but also the higher release rate difference between the corresponding condition and the non-therapeutic one. Furthermore, the incorporation of CHEMS has also the purpose of making these nanosystems fusogenic in the presence of the acidic pH of tumor microenvironment and the endosomes and lysosomes of tumor cells [48]. These results point to a lipid combination with excellent potential for targeted and controlled therapeutic action in tumor environments.

Concerning group C, the DPPC/DOPE formulation showed an irregular behavior from 21 h onwards, when the calculated % DOX release started to decrease. The results suggest that the DOX is taken up again by lipid structures that result from the inherent instability of these nanosystems. This is consistent with the stability assays of this formulation, which demonstrated the appearance of multiple-sized populations and a significant increase in PDI in the days after preparation. This instability is caused by the adoption of a non-bilayer structure by DOPE (hexagonal, H_II_) in an aqueous medium at neutral pH [69] and can be counteracted by adding CHEMS to the formulation, which helps the assembly. This is verified in the DOX release profile from DPPC/DOPE/CHEMS formulation, in which a regular release profile was verified. The addition of DSPE-PEG resulted in a substantial decrease in DOX release under all conditions. As the addition of DOPE resulted in the loss of thermosensitivity, the controlled release is ensured by the pH-dependent solubility of DOX and by the pH-induced destabilization of DOPE. The presence of PEG as a diffusion barrier protects the formulation from this pH sensitivity, which was also found in the previous formulations.

The DOX release kinetics of all the formulations and release conditions was fitted to three kinetic models: Weibull [37], first-order [38], and Korsmeyer-Peppas [39] (see Supplementary Materials, Tables S2–S4 for Group A, B, and C, respectively). The formal parameter b of Weibull model, besides characterizing the release kinetics curve type, is also associated with three different release diffusional mechanisms: (i) combined mechanism of Fickian diffusion and Case II transport when 0.75 < b < 1; (ii) Fickian diffusion (in either fractal or Euclidian spaces) when b ≤ 0.75; and (iii) complex release mechanism when b > 1. Likewise, the transport exponent (n) of the Korsmeyer-Peppas model also gives access to the type of release mechanism. When n < 0.45, the release mechanism is diffusion-controlled (Fickian release), 0.45 < n < 0.89 indicates a combination of diffusion and erosion drug release (non-Fickian release), 0.89 < n < 1 indicates a relaxation-controlled release, and in the case of n > 1, the release is controlled by swelling and chain relaxation.

In Group A, the addition of CHEMS to DPPC changed the mechanism of DOX release. In DPPC, there was a complex release mechanism at 42 °C, in both pH conditions, and a Fickian diffusion at 37 °C. However, the fit of release from DPPC/CHEMS to the Weibull model revealed, in all applied conditions, 0.75 < b < 1, that corresponds to diffusion in normal Euclidian substrate with the contribution of another release mechanism [37]. The fit to the Korsmeyer-Peppas model, although with lower *R*^2^ values, revealed values of *n* < 0.45, which are characteristic of a diffusion-controlled release mechanism (Fickian release). The release mechanism was changed again with the addition of DSPE-PEG. The DPPC/CHEMS/DSPE-PEG formulation only assumed good *R*^2^ values in the fit to the Weibull model, with b > 1 in all conditions studied, revealing a complex release mechanism.

In Group B, the fit of release from DPPC/DSPC to the Weibull model revealed a transversally b > 1, whose sigmoid curve indicates the action of a complex release mechanism. The DPPC/DSPC/CHEMS formulation demonstrated different types of release associated with each of the applied experimental conditions. For pH 5.5 and 42 °C, a b value of 0.59 was estimated, corresponding to diffusion in fractal or disordered substrate different from the percolation cluster [37], while at 37 °C, the b value is 1.1, corresponding to a complex mechanism. At pH 7.4 and 42 °C, a good *R*^2^ value was not found for any of the models, while at 37 °C, a b value of 0.38 reveals a diffusion mechanism in fractal substrate morphologically similar to the percolation cluster [70]. Like Group A, the addition of DSPE-PEG revealed a b > 1 in all conditions studied, indicating a complex release mechanism. Considering Group C, good *R*^2^ values were only obtained by fitting to the Weibull model. All formulations in this group showed b > 1 under all conditions, indicating that a complex mechanism governs the release of DOX in these formulations. This means a non-linear release rate increase in the initial phase up to the inflection point [37].

## 4. Conclusions

Liposomes are the colloidal nanocarriers with the highest potential as mediators for targeted and combined cancer therapy. Besides increasing the stability and bioavailability of drugs, the versatility in production with multiple lipid combinations and conjugating them with magnetic nanoparticles—forming magnetoliposomes—allows to enlarge the range of their applicability, targeting and combined therapies.

This work allowed confirming the reproducibility of the previously developed synthesis method for SMLs of different lipid combinations. Furthermore, all the synthesized SMLs exhibited hydrodynamic sizes and polydispersity indices required for biomedical applications. The high values of encapsulation efficiency (87% < EE% < 96%) point to these systems as advantageous DOX nanocarriers. It was proven the ability of SMLs to mask the positive charge of DOX, giving them the potential to avoid multi-drug resistance. The thermodynamic parameters of DOX-loaded SMLs revealed thermosensitivity for the DPPC- and DPPC/DSPC-based formulations (Groups A and B), with T_m_ of 41.34 ± 0.02 °C for DPPC/CHEMS/DSPE-PEG and T_m_ of 41.4 ± 0.2 °C for DPPC/DSPC/CHEMS/DSPE-PEG. In contrast, all formulations containing DOPE demonstrated a loss of thermosensitivity. The role of DSPE-PEG in lipid formulations should be highlighted. In addition to allow obtaining SMLs with optimal thermosensitivity for controlled release under mild-hyperthermia conditions, PEGylation proved to be fundamental as a long-term storage stabilizer, providing stealth properties under physiologically relevant conditions, which is a determining factor for the bioavailability of drugs at the target site.

Release profiles revealed a synergistic effect of a temperature rise (42 °C) with acidic pH (5.5), which are crucial for the controlled release of DOX. These profiles result from an interplay of the formulations’ thermosensitivity, the protonation of DOX at acidic pH, and its displacement to SMLs surface. Once again, formulations based on DPPC and DPPC/DSPC (Groups A and B, respectively) stand out as multi-stimuli-responsive systems (thermo/pH sensitivity) under therapeutic conditions (42 °C and/or acidic pH). Although DSPE-PEG acts as a DOX diffusion barrier, PEGylated formulations, particularly the DPPC/DSPC/CHEMS/DSPE-PEG, demonstrate an overall potential for a targeted and controlled therapeutic action in tumor environments.

## Figures and Tables

**Table 1:** Lipid composition and molar ratio of the lipids to synthesize SMLs with different types of action.

Group	Lipid Composition	Molar Ratio	Type
A	DPPC	1	Non-long-circulating and thermosensitive
	DPPC/CHEMS	9:1	Non-long-circulating, fusogenic, thermo- and pH-sensitive
	DPPC/CHEMS/DSPE-PEG	80:15:5	Long-circulating, fusogenic, thermo- and pH-sensitive
B	DPPC/DSPC	3:1	Non-long-circulating and thermo-sensitive
	DPPC/DSPC/CHEMS	7:2:1	Mid-long-circulating, fusogenic thermo- and pH-sensitive
	DPPC/DSPC/CHEMS/DSPE-PEG	60:20:15:5	Long-circulating, fusogenic, thermo- and pH-sensitive
C	DPPC/DOPE	7:3	Non-long-circulating, fusogenic, pH-sensitive
	DPPC/DOPE/CHEMS	6:3:1	Non-long-circulating, fusogenic, pH-sensitive
	DPPC/DOPE/CHEMS/DSPE-PEG	50:30:15:5	Long-circulating, fusogenic, pH-sensitive

**Table 2:** Hydrodynamic diameter (D_H_), polydispersity index (PDI), transition temperature and DOX encapsulation efficiency (EE%) of DOX-loaded SMLs based on different lipid formulations at 25 °C.

Group	Lipid Composition	D_H_ (nm)	PDI	EE%	Isoelectric pt.	T_m_ (°C)
A	DPPC	116 ± 3	0.18 ± 0.02	96.9 ± 0.5	7.80	41.40 ± 0.02
	DPPC/CHEMS	149 ± 7	0.21 ± 0.01	95 ± 1	5.61	38.2 ± 0.2
	DPPC/CHEMS/DSPE-PEG	156 ± 18	0.20 ± 0.02	94 ± 3	6.22	41.34 ± 0.02
B	DPPC/DSPC	157 ± 1	0.22 ± 0.03	88 ± 2	5.95	42.4 ± 0.2
	DPPC/DSPC/CHEMS	160 ± 1	0.20 ± 0.01	92 ± 2	5.37	39.7 ± 0.1
	DPPC/DSPC/CHEMS/DSPE-PEG	187 ± 4	0.22 ± 0.02	92 ± 5	5.61	41.4 ± 0.2
C	DPPC/DOPE	111 ± 17	0.24 ± 0.07	96.7 ± 0.5	8.00	*
	DPPC/DOPE/CHEMS	118 ± 4	0.20 ± 0.04	87 ± 12	5.49	*
	DPPC/DOPE/CHEMS/DSPE-PEG	117.39 ± 0.03	0.23 ± 0.06	93 ± 4	5.13	*

* T_m_ peak not found.

**Table 3:** Summary of release slope (*m*) values of DOX plotted in the first 5 h and ratio between the saturation release under 37 °C, pH 7.4 and the corresponding release conditions.

Group	Lipid Composition	42 °C, pH 5.5		42 °C, pH 7.4		37 °C, pH 5.5		37 °C, pH 7.4	
		*m*	Ratio	*m*	Ratio	*m*	Ratio	*m*	Ratio
A	DPPC	4.04	5.67	1.77	1.47	1.30	2.21	0.68	1
	DPPC/CHEMS	6.39	1.91	2.58	1.12	5.92	1.25	2.70	1
	DPPC/CHEMS/DSPE-PEG	4.71	2.30	2.39	1.54	3.53	1.74	2.52	1
B	DPPC/DSPC	6.39	1.86	2.32	0.59	5.89	1.12	2.7	1
	DPPC/DSPC/CHEMS	4.23	2.88	1.77	1.51	5.25	2.92	0.98	1
	DPPC/DSPC/CHEMS/DSPE-PEG	7.21	1.75	1.74	0.93	6.19	1.71	2.11	1
C	DPPC/DOPE	4.04	2.48	2.70	2.01	4.20	1.88	0.79	1
	DPPC/DOPE/CHEMS	5.83	2.42	3.26	1.48	3.15	1.37	2.67	1
	DPPC/DOPE/CHEMS/DSPE-PEG	3.42	1.66	1.69	0.93	1.83	1.82	1.03	1

## Data Availability

Not applicable.

## Supplementary Materials

The following supporting information can be downloaded at: https://www.mdpi.com/article/10.3390/biomedicines10051207/s1, Table S1: Dissociation constant ($k_{d}$), binding constant ($k_{b}=\frac{1}{k_{d}}$) and number of binding locations (n) of liposomes to HSA; Table S2: Obtained constant values by the fitting of each mathematical model to the kinetic data and respective coefficient of determination (*R*^2^), according to the temperature and pH variation for Group A lipid formulations; Table S3: Obtained constant values by the fitting of each mathematical model to the kinetic data and respective coefficient of determination (*R*^2^), according to the temperature and pH variation for Group B lipid formulations; Table S4: Obtained constant values by the fitting of each mathematical model to the kinetic data and respective coefficient of determination (*R*^2^), according to the temperature and pH variation for Group C lipid formulations.
