# Supplementary material for: Solid Magnetoliposomes as Multi-Stimuli-Responsive Systems for Controlled Release of Doxorubicin: Assessment of Lipid Formulations
Source: Biomedicines. 2022 May 23;10(5):1207. doi: 10.3390/biomedicines10051207 (PMC9138220; doi:10.3390/biomedicines10051207)
Supplement: Supplementary file 1 [file biomedicines-10-01207-s001.zip › biomedicines-1728169-supplementary.pdf]

Solid magnetoliposomes as multi-stimuli-responsive systems for controlled release of doxorubicin: assessment of lipid formulations

Beatriz D. Cardoso, Vanessa F. Cardoso, Senentxu Lanceros-Méndez and Elisabete M. S. Castanheira

Supplementary material

1. Interaction with Human Serum Albumin

The calculated variables (following Equation 3) for the interaction of DOX-loaded SMLs with Human Serum Albumin (HSA) are summarized in Table S1.

Table S1. Dissociation constant (k_d), binding constant ($k_b = \frac{1}{k_d}$) and number of binding locations (n) of liposomes to HSA.

		k_d (M)	k_b (M^{-1})	n	R^2
**Group**	Free DOX	8.24×10^{-7}	5.26×10^{6}	1.80	0.96
**A**	DPPC	2.00×10^{-7}	5.00×10^{6}	1.60	0.96
	DPPC/ CHEMS	1.21×10^{-6}	8.25×10^{5}	1.53	0.98
	DPPC/ CHEMS/DSPE-PEG	1.72×10^{-6}	5.81×10^{5}	1.44	0.97
**B**	DPPC/DSPC	9.22×10^{-7}	1.01×10^{6}	1.67	0.99
	DPPC/DSPC/CHEMS	4.27×10^{-7}	2.12×10^{6}	1.66	0.99
	DPPC/DSPC/CHEMS/DSPE-PEG	8.16×10^{-7}	1.23×10^{6}	1.53	0.98
**C**	DPPC/DOPE	7.72×10^{-7}	1.30×10^{6}	1.62	0.98
	DPPC/DOPE/CHEMS	2.92×10^{-7}	3.43×10^{6}	1.48	0.98
	DPPC/DOPE/CHEMS/DSPE-PEG	9.90×10^{-7}	1.01×10^{6}	1.46	0.98

2. Drug release kinetics and mathematical modelling of the release profile

The Weibull model is expressed by Equation S.1 in terms of the drug fraction accumulated (m) in solution at the time t [1]:

$$m = 1 - exp^{-(t-T_i)^{\frac{b}{a}}} \quad \text{(S.1)}$$

where a is a scale parameter that defines the timescale of the process, T_i represents the latency time of the release process (often being zero), and b is a parameter that characterizes the type of curve (b = 1 is

exponential; $b > 1$ is sigmoid, with ascendant curvature delimited by an inflection point; and $b < 1$ is parabolic, displaying high initial slope and a consistent exponential character).

The first-order kinetic model is described by Equation S.2 [2]:

$$F(\%) = M_0 \times (1 - e^{-kt}) \tag{S.2}$$

where F(%) is the percentage of released drug, M_0 represents the total amount of the drug released, k represents the first-order rate constant and t the time. Considering that the total drug release varies between experiments, M_0 was considered as a variable.

The Korsmeyer–Peppas model (power law) is a more comprehensive semi-empirical equation that establishes an exponential relationship between release and time, following Equation S.3 [3]:

$$C_t / C_0 = K \cdot t^n \tag{S.3}$$

where C_0 and C_t are the concentrations at time 0 and t, respectively, K is the rate constant and n is the transport exponent.

The constants values and coefficients of determination obtained for each model are summarized in Table S2 (for DPPC-based SMLs), Table S3 (for DPPC/DSPC-based SMLs) and Table S4 (for DPPC/DOPE-based SMLs).

Table S2. Parameters obtained by the fitting of the different mathematical model for the kinetic data and corresponding coefficient of determination (R^2), according to the temperature and pH variation for Group A lipid formulations.

Group A											
	pH	T	y_{max}(%) ± SD	Weibull			First-order		Korsmeyer-Peppas		
				b	*a*	R^2	*k*	R^2	*K*	*n*	R^2
DPPC	5.5	42 °C	25 ± 2	1.18	0.29	0.99	0.35	0.99	10.76	0.30	0.88
		37 °C	9 ± 1	0.89	0.37	0.94	0.34	0.93	3.74	0.28	0.90
	7.4	42 °C	6.5 ± 0.2	1.59	0.71	0.96	0.82	0.94	4.66	0.14	0.74
		37 °C	4 ± 1	0.83	0.34	0.88	0.27	0.88	2.64	0.28	0.83
DPPC/CHEMS	5.5	42 °C	35 ± 5	0.99	0.61	0.98	0.61	0.98	23.84	0.15	0.92
		37 °C	21.1 ± 0.3	0.77	0.72	0.91	0.65	0.91	13.90	0.15	0.89
	7.4	42 °C	23 ± 1	0.75	0.28	0.98	0.23	0.97	5.90	0.37	0.94
		37 °C	18 ± 1	0.92	0.32	0.97	0.31	0.97	7.46	0.29	0.94
DPPC/CHEMS/ DSPE-PEG	5.5	42 °C	25 ± 2	4.60	0.01	0.94	0.30	0.83	9.74	0.36	0.65
		37 °C	16 ± 2	2.44	0.07	0.98	0.29	0.87	5.94	0.33	0.70
	7.4	42 °C	17 ± 2	1.53	0.14	0.86	0.25	0.84	4.61	0.36	0.76
		37 °C	11 ± 5	10.0	8.23×10^{-5}	0.97	0.38	0.58	5.51	0.24	0.35

Table S3. Fitting parameters for each mathematical model for the kinetic data and corresponding coefficient of determination (R^2), according to the temperature and pH variation for Group B lipid formulations.

**Group B**											
	**pH**	**T**	y_{max}**(%) ± SD**	**Weibull**			**First-order**		**Korsmeyer-Peppas**		
				b	*a*	R^2	*k*	R^2	*K*	*n*	R^2
**DPPC/DSPC**	5.5	42 °C	40 ± 3	2.11	0.08	0.98	0.30	0.91	14.50	0.31	0.70
		37 °C	24.1 ± 0.9	21.97	1.99	0.98	0.90	0.74	0.50	0.30	0.42
	7.4	42 °C	12.8 ± 0.6	1.57	0.18	0.97	0.35	0.94	6.06	0.27	0.69
		37 °C	14 ± 2	1.66	0.35	0.98	0.85	0.60	12.11	0.19	0.47
**DPPC/DSPC/ CHEMS**	5.5	42 °C	25 ± 1	0.59	0.55	0.94	0.51	0.64	12.12	0.20	0.96
		37 °C	25.7 ± 0.5	1.11	0.48	0.97	0.45	0.36	19.80	0.10	0.93
	7.4	42 °C	13 ± 1	0.06	0.02	0.74	0.13	0.18	8.89	0.05	0.72
		37 °C	8 ± 1	0.38	0.37	0.98	0.18	0.73	0.24	3.5	0.97
**DPPC/DSPC/ CHEMS/DSPE-PEG**	5.5	42 °C	22 ± 3	9.872	0.001	0.97	0.19	0.44	12.83	0.19	0.44
		37 °C	21 ± 1	4.456	0.005	0.95	0.43	0.73	5.86	0.43	0.73
	7.4	42 °C	11 ± 1	1.39	0.15	0.86	0.36	0.78	3.33	0.36	0.78
		37 °C	12 ± 1	1.72	0.15	0.88	0.49	0.92	3.83	0.49	0.92

Table S4. Fitting parameters for each mathematical model to the kinetic data and corresponding coefficient of determination (R^2), according to the temperature and pH variation for Group C lipid formulations.

**Group C**											
	**pH**	**T**	y_{max}**(%) ± SD**	**Weibull**			**First-order**		**Korsmeyer-Peppas**		
				b	*a*	R^2	*k*	R^2	*K*	*n*	R^2
**DPPC/DOPE**	5.5	42 °C	21 ± 1	6.747	0.001	0.98	0.31	0.76	8.79	0.29	0.55
		37 °C	16 ± 2	7.53	6.44×10^{-5}	0.94	0.28	0.67	7.57	0.29	0.45
	7.4	42 °C	17 ± 1	1.68	0.17	0.91	0.31	0.87	6.84	0.28	0.71
		37 °C	8 ± 1	1.68	0.03	0.91	0.08	0.88	1.45	0.64	0.84
**DPPC/DOPE/ CHEMS**	5.5	42 °C	25 ± 2	1.91	0.32	0.99	0.69	0.99	21.2	0.06	0.95
		37 °C	14.6 ± 0.8	1.32	0.23	0.78	0.33	0.77	8.75	0.21	0.73
	7.4	42 °C	15.7 ± 0.3	3.30	0.03	0.97	0.35	0.90	8.57	0.19	0.81
		37 °C	10 ± 2	10.47	0.001	0.96	0.40	0.76	7.61	0.16	0.65
**DPPC/DOPE/ CHEMS/DSPE-PEG**	5.5	42 °C	22 ± 3	9.87	0.001	0.97	0.32	0.69	12.83	0.19	0.44
		37 °C	21 ± 2	4.45	0.005	0.95	----	----	5.86	0.43	0.73
	7.4	42 °C	11 ± 1	1.39	0.15	0.85	----	----	3.35	0.36	0.78
		37 °C	18 ± 1	1.54	0.07	0.98	----	----	3.83	0.49	0.92

References

1. Noyes, A.A.; Whitney, W.R. The rate of solution of solid substances in their own solutions. *Journal of the American Chemical Society* **1897**, *19*, 930-934. doi: 10.1021/ja02086a003
2. Papadopoulou, V.; Kosmidis, K.; Vlachou, M.; Macheras, P. On the use of the Weibull function for the discernment of drug release mechanisms. *International Journal of Pharmaceutics* **2006**, *309*, 44-50. doi:10.1016/j.ijpharm.2005.10.044
3. Korsmeyer, R.W.; Gurny, R.; Doelker, E.; Buri, P.; Peppas, N.A. Mechanisms of solute release from porous hydrophilic polymers. *International Journal of Pharmaceutics* **1983**, *15*, 25-35. doi:10.1016/0378-5173(83)90064-9
